# Characterization of the complete chloroplast genome of Taibaisanqi (*Tongoloa silaifolia*)

**DOI:** 10.1080/23802359.2019.1661299

**Published:** 2019-09-06

**Authors:** Ling-Jian Gui, Deng-Feng Xie, Sheng-bin Jia, Hao Li, Yan-Ping Xiao, Xing-Jin He

**Affiliations:** Key Laboratory of Bio-Resources and Eco-Environment of Ministry of Education, College of Life Sciences, Sichuan University, Chengdu, China

**Keywords:** Apiaceae, chloroplast genome, Illumina sequencing, phylogenetic analysis

## Abstract

*Tongoloa silaifolia*, known as a traditional Chinese medicine Taibaisanqi, is a perennial herb of Apiaceae. In this study, the complete chloroplast genome of *T. silaifolia* was determined through Illumina sequencing method. The chloroplast genome of *T. silaifolia* was 161,122 bp in length and contained a pair of IR regions (30,824 bp) separated by a small single-copy region (17,553 bp) and a large single-copy region (81,921 bp). This chloroplast genome is encoded with 137 genes including 92 CDS, 37 tRNA genes, and 8 rRNA genes. The overall GC content of *T. silaifolia* cp genome is 37.7%. By phylogenetic analysis using maximum likelihood method, *T. silaifolia* showed the closest relationship with *Chuanminshen violaceum* and *Hansenia* species.

*Tongoloa silaifolia* (de Boiss.) Wolff, a perennial herb of Apiaceae, commonly known as “Taibaisanqi” in Shaanxi, China, is a traditional Chinese medicine. According to traditional medicine theory, the root has the functions of removing blood stasis, stopping bleeding, strengthening bones, and muscles (SATCM 1999). Chemical composition analysis showed that *T. silaifolia* contains coumarins, flavonoids, sterols, and other compounds ( O'Kennedy and Thornes 1997, Qin et al. 2013 ). With an aim to retrieve valuable cp molecular markers, indels, and SSRs by comparative analyses with other Apiaceae chloroplast (cp) genomes, we assembled and analyzed the complete cp genome of *T. silaifolia* based on the next-generation sequencing method.

The leaves of *T. silaifolia* were collected from Taibai Mountain (Shaanxi, China), the specimens were deposited in Sichuan University Herbarium (SZ), and the voucher number is LH2018072802. The whole genome sequencing was achieved on the Illumina Hiseq Platform (Illumina, San Diego, CA, USA). The complete cp genome was assembled via NOVOPlasty (Dierckxsens et al. 2017) and then annotated using PGA-master (Qu et al. 2019) with the cp genome annotations of *Amborella trichopoda* (GenBank number AJ506156) and *Hansenia forbesii* (GenBank number NC_035054) as references. Manual proofreading was performed in Geneious version 10.2 (Kearse et al. 2012). The complete cp genome was then submitted to GenBank with the accession number MN218688.

The cp genome of *T. silaifolia* is a typical quadripartite structure with a length of 161,122 bp, containing a pair of IR regions (30,824 bp) separated by a small single-copy region (17,553 bp) and a large single-copy region (81,921 bp). The cp genome possesses 137 genes, including 92 protein-coding genes (CDS), 8 rRNA genes, and 38 tRNA genes. The overall GC content of the cp genome is 37.7%.

To ascertain the phylogenetic relationship between *T. silaifolia* and other Apiaceae taxa, 13 complete cp genomes of 11 genera were achieved from the National Center for Biotechnology Information (NCBI). A maximum likelihood (ML) tree with 200 bootstrap replicates (Figure 1) was constructed using RAxML v8 (Stamatakis 2014). The result shows that *T. silaifolia* located at the base of Apioideae is closely clustered with *Chuanminshen violaceum* and *Hansenia* species, which validates the previous studies (Zhou et al. 2009, Downie et al. 2010). The complete cp genome provides useful materials for further phylogenetic and evolutionary studies of *Tongoloa*.

**Figure 1.**
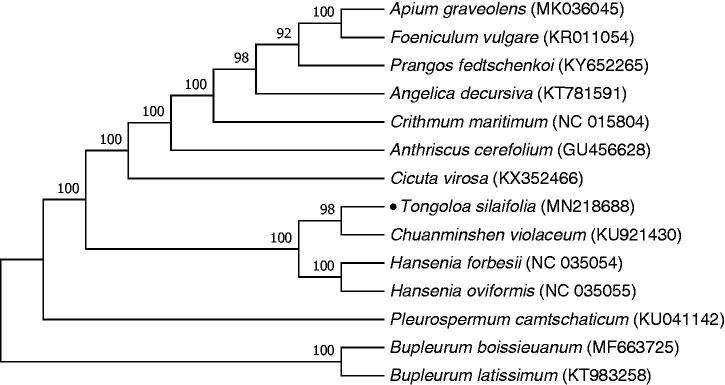
ML phylogenetic tree of T. silaifolia with 13 species of Apiaceae was constructed by complete cp genome sequences. Numbers above the branches are bootstrap values.
